# Biopharmaceutics 4.0, Advanced Pre-Clinical Development of mRNA-Encoded Monoclonal Antibodies to Immunosuppressed Murine Models

**DOI:** 10.3390/vaccines9080890

**Published:** 2021-08-11

**Authors:** Andreas Ouranidis, Theodora Choli-Papadopoulou, Eleni T. Papachristou, Rigini Papi, Nikolaos Kostomitsopoulos

**Affiliations:** 1 Department of Pharmaceutical Technology, School of Pharmacy, Aristotle University of Thessaloniki, 54124 Thessaloniki, Greece; 2 Department of Chemical Engineering, Polytechnic School, Aristotle University of Thessaloniki, 54124 Thessaloniki, Greece; 3 Department of Chemistry, Aristotle University of Thessaloniki, 54124 Thessaloniki, Greece; tcholi@chem.auth.gr (T.C.-P.); epapachristou@chem.auth.gr (E.T.P.); rigini@chem.auth.gr (R.P.); 4 Center of Clinical, Experimental Surgery and Translational Research, Biomedical Research Foundation of the Academy of Athens, 11527 Athens, Greece; nkostom@bioacademy.gr

**Keywords:** mRNA encoded antibodies, SARS–CoV-2, COVID-19, Trastuzumab, mRNA-vaccines, pharmacokinetics, immunodeficient, NOD/SCID/J, biopharmaceutics 4.0, IVT mRNA

## Abstract

Administration of mRNA against SARS-CoV-2 has demonstrated sufficient efficacy, tolerability and clinical potential to disrupt the vaccination field. A multiple-arm, cohort randomized, mixed blind, placebo-controlled study was designed to investigate the in vivo expression of mRNA antibodies to immunosuppressed murine models to conduct efficacy, safety and bioavailability evaluation. Enabling 4.0 tools we reduced animal sacrifice, while interventions were designed compliant to HARRP and SPIRIT engagement: (a) Randomization, blinding; (b) pharmaceutical grade formulation, monitoring; (c) biochemical and histological analysis; and (d) theoretic, statistical analysis. Risk assessment molded the study orientations, according to the ARRIVE guidelines. The primary target of this protocol is the validation of the research hypothesis that autologous translation of Trastuzumab by in vitro transcribed mRNA-encoded antibodies to immunosuppressed animal models, is non-inferior to classical treatments. The secondary target is the comparative pharmacokinetic assessment of the novel scheme, between immunodeficient and healthy subjects. Herein, the debut clinical protocol, investigating the pharmacokinetic/pharmacodynamic impact of mRNA vaccination to immunodeficient organisms. Our design, contributes novel methodology to guide the preclinical development of RNA antibody modalities by resolving efficacy, tolerability and dose regime adjustment for special populations that are incapable of humoral defense.

## 1. Introduction

Although equipped with a finite gene repertoire, the human immune system effectively encounters numerous pathogens and antigens over the lifespan cycle, by generating fit-to-purpose immunoglobulins [1]. B-immune cells endeavor this mission by executing a unique set of bioprocesses, i.e., splicing, gene segment recombination and random nucleotide insertion in order to form the B-cell receptors, which in turn, pass through elimination selection drill of self-reactive and nonproductive specificities [2], in order to produce the heavy and light immunoglobulin chains. The latter, self-assemble to deliver a mature, non-self-reactive antigen recognition molecular machinery, eventually transformed to the secreted antibody structure [3].

In particular, the IgG of the antibody isotypes is the one comprising the majority of serum immunoglobulins and features the greatest stability [4]. The human and mouse IgG subclasses present morphological and functional similarities, both being suitable for neutralizing toxins and viruses [5]. Moreover, they demonstrate participation activity similarities towards secondary responses, whilst opsonization occurs when the variable antibody portion (Fab) binds to the cell membrane target, thus, forming the immune complex. Neutrophils and monocytes recognize the Fc antibody region using their activating receptors and induce T-cell responses promoted by the MHC antigen presentation responsible for the pathogen elimination [6]. Antibodies are recognized by the expressed NK cell Fc receptor, bound to pathogens, encompassing the antibody-dependent cell-mediated cytotoxicity (ADCC), releasing cytokines and effecting cellular apoptosis [7].

A monoclonal antibody (mAb) is an artificial, antigen-specific immunoglobulin, derived by the cloning of a unique parent white blood cell [8]. Monoclonal antibodies have been excessively used to treat cancers with proven safety. In particular, the recombinant, humanized, Trastuzumab (TZM) IgG1 class monoclonal antibody, binds selectively to the extracellular domain of the human epidermal factor receptor 2 (HER2), inhibiting the signaling pathways, reducing the mechanism of angiogenesis [9]. It was recently showed experimentally and with advanced numeric tools that the HER2–Trastuzumab interaction is much more heterogeneous than understood before, with two lumped pairs of kd and ka, respectively [10]. Marketed TZM is a sterile, pale yellow, lyophilized, preservative-free powder, reconstituted in bacteriostatic water solution prior to intravenous administration, authorized by the EMA and FDA regulatory bodies for the management and/or treatment of HER2 neu-positive breast (BC) and gastric (GC) cancer [11]. The recommended infusion weight-based dose regime emancipates 8 mg/kg loading followed by 6 mg/kg of maintenance protocols, administrated every three to four weeks [12]. Trastuzumab pharmacokinetics have been found to exhibit both linear and nonlinear elimination, the latter being attributed to mediated drug disposition [13]. At a steady state, a total clearance rate and a half-life terminal elimination period of 0.24 L/h and 30 days, have been respectively observed [14]. The large scale production of TZM features intrinsic complexities namely the aberrant post-translational modifications, tedious purification protocols, quality control hurdles, mammalian antibody-producing culture mutations and non-passage stabilization events [15]. Moreover, TZM mAbs appear oversensitive to clearance mechanisms and enzymic serum degradation, thus, requiring frequently repeated dosing, i.e., an intervention scheme that augments the risk of unwanted adverse effects such as immunologic activation [16], while also increasing the related health system expenditure. Alternatively, TZM antibodies may be designed as software source code under the enabling 4.0th industrial age computation tools [17], and are produced on demand by the preparation of in vitro transcribed (IVT) mRNA [18].

The latter, has demonstrated the potential to deliver translational therapeutic concentration levels of proteinic constructs and superiority of safety when compared to viral vectors and/or DNA plasmid transfection, due its transient nature [19]. Elaborating on this, the risk of insertional mutagenesis is insignificant with IVTs, hence the iterated mRNA code fragments never penetrate the cell nucleus [18]. In addition, IVT mRNA does not require the cumbersome handling of eukaryotic cultures, presents uniform quality, while the autologous translation pathway ameliorates the risk of any inappropriate post-translational modifications in a biomimetic fashion [20]. Expanding, the IVT mRNA allows for the natural glycosylation associated to the Fc immunoglobulin domain, which critically affects the antibody function. The N-linked site of glycosylation is located at Asn297 on both -CH_2_ domains, forming a biantennary heptasaccharide consisting of mannose and N-acetylglucosamine [21]. Functional variations of terminal galactose, sialic acid, N-acetylglucosamine and core fucosylation are located within the two glycosylation sites leading to 32 possible patterns. The glycans interact with the hydrophobic pocket of the Fc domain stabilizing the immunoglobulin [22]. Glycans influence the binding to the Fc receptors of immune mediators and effector cells. N-glycan at the conserved Asn297 locus has been pointed as critical for the binding of IgG with FcγR making significant contribution to in vivo functionality. Antibody-dependent cellular cytotoxicity (ADCC) mechanism of coated antigens activates the monocytes to bind on the FcγR complex [23]. Moreover the complement dependent cytotoxicity (CDC), dependent of glycosylation events, requires the presence of a complex domain containing at least two N-acetylglucosamines and multiple sialic acids and galactoses [24].

The humoral, thymus-dependent immune responses are interlinked with the immunoglobulin-mediated antigen recognition and binding to Ig receptors of B-cells, fueling the consequent intracellular signaling transduction [25]. The decomposition signal of the C3 supplement initiates a second, meta-signal, activating the clonal expansion of the B-cells, inducing the somatic mutations of the immunoglobulin V domains of the gene repertoire. The iterated natural mechanism, is circumvented in the case of IVT-mRNA encoded antibodies, whereas the biosynthesis of the latter is attributed to the autologous ribosomal translation process [18]. This response follows the provision of the mRNA poly-peptidic decoding which in turn circumvents the natural immune pathway order. Immunosenescence is a state of inability of the immune system, prevalent to elderly patients, according to which B-cell responses, antibody titres and cytokine production become reduced. For such special patient populations, the administration of mRNA encoded antibodies might offer improved treatment efficacy.

Indeed, under this scientific hypothesis, apart from the lymphocytes, a wide variety of unchartered cell species may also function as alternative bioreactors of immunoglobulin synthesis. Contemplating the scenario that these in vivo produced immunoglobulins are structurally complete and functionally capable, our findings would aid the discovery of a novel pharmacokinetic and pharmacodynamic profile of antibody bioavailability for immunodeficient subjects. Given the wide application of the mRNA technology to several of diseases, including anti COVID-19 vaccination [26], our experimental efforts would contribute usefully, both basic and applied, in research.

Therefore, the primary target of the presented pre-clinical trial protocol, is the experimental validation of the research scenario that the autologous decoding of antibodies via administration to immunosuppressed animal models, being incapable of producing their natural antibodies, is non-inferior when compared to classical TZM protein treatment. In line with this, the escorting measurements taking place include the quantification of biodistribution IVT-mRNA encoded antibodies and their carriers via histological microscopy. Furthermore, a basic metabolic, toxicological and inflammation panel of blood markers shall elucidate the corresponding adverse effects and the systemic biodistribution of the test drug substance. The secondary target refers to a PK comparative assessment of the novel drug treatment between immunodeficient strains versus healthy populations. The pilar pharmacokinetic measurements amongst others that will take place for both PK related arms are the area under the curve (AUC), maximum concentration (C_max_), time T_max_ of accomplished C_max_, half-life (t_1/2_) and maximum observed response (E_max_).

## 2. Materials and Methods

### 2.1. Recruitment and Animal Justification towards Future Extrapolation to Human Populations 

NOD.CB17-*Prkdc^scid^*/J (NOD/SCID/J) immunodeficient inbred [27,28] and C57BL/6J [29] mice will be purchased from Charles River (https://www.criver.com/products-services/find-model/jax-nod-scid-mice?region=3616, accessed on 2 August 2021), France and will be maintained in the animal facility of the Biomedical Research Foundation of the Academy of Athens under Specific Pathogen Free (SPF) conditions. Mice will enter to the experiments at the age of 10–21 weeks old. All mice will be caged in individual ventilated cages (BlueLine Next, Tecniplast, Buguggiate, Italy) under controlled room conditions supplied with HEPA-filtered air at 15 Air Changes per Hour (ACH) at a temperature of 22 ± 2 °C and relative humidity 55 ± 10%, a 12:12 h light: dark cycle (lights on at 0700), light intensity of 300 lux at one meter above the floor in the middle of the room, and positive air pressure of 0.6 Pa within the room. Room conditions are continuously monitored through the central Building Management System (BMS) of the animal facility. All mice will have ad libitum access to filtered tap water in drinking bottles and a pelleted chow that contained 18.5% protein, 5.5% fat, 4.5% finer, and 6% ash (4RF22, Mucedola, Milan, Italy). The bedding in each cage of the three housing systems will be comprised of autoclaved corncob bedding (240 g; Rehofix MK 2000, J. Rettenmaier and Sons, Rosenberg, Germany) while cage and bedding changes will be made once per week. Dirty cages of all groups will be changed under a cage change station (Interactive SafeChange™, Tecniplast, Buguggiate, Italy). The choice of NOD/SCID/J mice mirrors the pre-clinical trial’s directive to perform the experiments on a severely immunodeficient phenotype [30]. The latter, refers to a lack of both functional B- and T- lymphocytes, either due to the Prkdc (protein kinase DNA activated, catalytic polypeptide: DNA-PKCs) deletion or to the absence of variable (V)-diversity (D)-joining (J) (V(D)J recombination [31]. The selected animal model carries multiple, precious for our study, abnormalities of the immune system, including the loss of impaired and complement NKs, dendritic cells and macrophage functions [32]. Moreover, NOD/SCID/J strain, in particular, are insulitis- and diabetes- free throughout life span and therefore none of exogenous inflammation incidents are prone to disrupt the credibility of the model [33]. Consequently, the NOD/SCID/J animal model is justified as our preferred animal type, to evidently serve as the study investigation model, hence, it fully meets the requirements of the in vivo mAb pharmacometric study of functional immunoglobulins being generated by non b- lymphocytic cell factories. In addition, the C57BL/6/J mouse ascends as the coupled model of choice, fulfilling the scope of the regression comparative analysis control, hence, exhibiting the nearest possible ancestral affinity to simulate the healthy physiological immune system. Only male murine models will be recruited in a single round draft reflecting our efforts to avoid any unwanted physiological sex dependent variance on the pharmacokinetic and biochemical corresponding variables investigated [34].

### 2.2. Study Design and Setting

A two-month, observational non-inferiority multiple-arm and stage cohort randomized, premier, double mixed blind placebo-controlled, single-centered trial was thoroughly designed to investigate the in vivo expression of mRNA encoded TZM monoclonal antibodies in immunosuppressed animal models and drive extrapolating efficacy, safety and pharmacokinetic conclusions. The study falls to the sector of translational, precision oncology medicine of novel mRNA modalities and formulations, aiming to combat the human condition of breast and gastric HER2 positive cancer and extrapolate the pharmacokinetic and pharmacodynamic behavior, for the said special human populations. For this purpose, each animal of the engagement is considered as the experimental work unit of the study, taking into assessment a dropout rate of ≤10%. We proceed towards the enrollment of total 168 mice subjects in our accredited clinical cite, under the acceptance and monitoring of the relevant veterinary and bioethics committee. This study is designed in accordance with the latest of the veterinary and GCP guidelines for human medicine [35], namely the “International Cooperation on Harmonization of Technical Requirements for Registration of Human Products” and the “GL9 Good Clinical Practices” [36], the European Agency for the Evaluation of Medicinal Products (EMEA) “Guideline on statistical principles for veterinary clinical trials”, EMEA/CVMP/816/00-FINAL [37]. The study has been approved by the Institutional Project Evaluation Committee on the 26/02/2021 (Ref. Num. 148/26-02-2021), by the Directorate of Veterinary Department of Helenic Republic (272243/07-04-2021.300627) and strictly follows the (ARRIVE) ‘Animal Research: Reporting in-Vivo Experiments’ protocol guidelines’ [38] as they have been updated [39] for future human use. 

**Figure 1:**
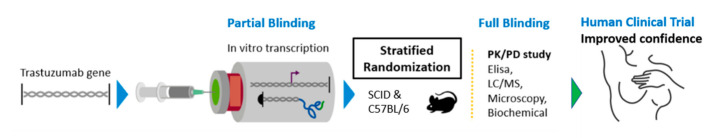
Schematic overview of the preparation and clinical validation of TZM antibodies encoded by mRNA.

The study is single centered, the work packages including the preparation of the drug doses as well as the clinical validation and analysis will be conducted by a control access SPF accredited facility, namely the Laboratory Animal Facility of the Biomedical Research Foundation of the Academy of Athens (BRFAA). This model study, as demonstrated by Figure 1, utilizes enabling modelling tools of the 4.0 industrial age to reduce animal sacrifice, while research actions are performed at multiple levels of engagement to ensure extrapolating results for future human trial protocols: (a) Randomization, blinding, decoding (b) drug formulation, administrations conduct and monitoring; (c) outcome measurements, i.e., biological, histological and chemical analysis documentation; (d) theoretical explanation of results; and (e) statistical analysis and final reporting.

### 2.3. Experimental Protocol, Animal Handling and Data Collection

All murine models are housed in accordance to the European Directive 2010/63 and the “Guide for the Care and Use of Laboratory Animals” [40]. A Building Managing System (BMS), monitors the electromechanical equipment of the facility, 24 h per day and documents hazardous alterations. The murine models recruited belong to either A or B cohort, namely the C57BL/6/J and NOD/SCID/J type respectively and are further taxonomized into the following intervention groups in order to fulfill the clinical study design: (a) A1, B1 of negative control that consists of 2 mice per type that will receive (*n* = 4) parenteral concentrate natural saline solution, (b) A2, Β2 of placebo control that consists of 2 mice per type (*n* = 4), placebo being a substance of identical visual characteristics, (c) A3, Β3 that consists of 36 mice per type (*n* = 72) receiving the carrier and IVT-mRNA encoding for the active pharmaceutical ingredient TZM, (d) A4, Β4 positive control that consists of 36 mice per type (*n* = 72) receiving the API TZM with the commercial carrier Herceptin, (e) A5, B5 that consists of 4 mice per type (*n* = 8) receiving mRNA GFP to guide the comparative tissue study, (f) A6, B6 that consists of 4 mice per type (*n* = 8) receiving protein GFP as positive control.

The day of the experimental administrations, all murine models shall have access to water and remain on fasted state for at least 8 h before infusion. The total infusion dose of the mRNA modality or control or signal indicative protein will for all groups be in concentrations that correlate to the established antibody, weight-based dose regime of 2 mg/kg. The total volumetric dose of the protein and carrier will be set at 100 μL in concentrations that are in line with the established antibody weight-based dose regime of 8 mg/kg. Pericardiocentesis of the animal subjects will be implemented towards the collection of blood samples (0.5–0.7 mL) for the time intervals of *t* = 12 days for the groups A1 and Β1, *t* = 12, 24 days for the groups A2 and Β2, *t* = 0.5, 1.0, 1.5, 2, 3, 4, 5, 6, 7, 12, 24, 28 days for the groups A3, Β3, A4 and Β4. The groups A5, B5 and A6, B6 will receive histopathological examination at the time points *t* = 6, 18, 24, 28 days in order to quantify the biodistribution of the mRNA to the several tissues after intravenous administration. The designed intervention groups under the HARRP guideline are presented by Table 1.

**Figure 2:**
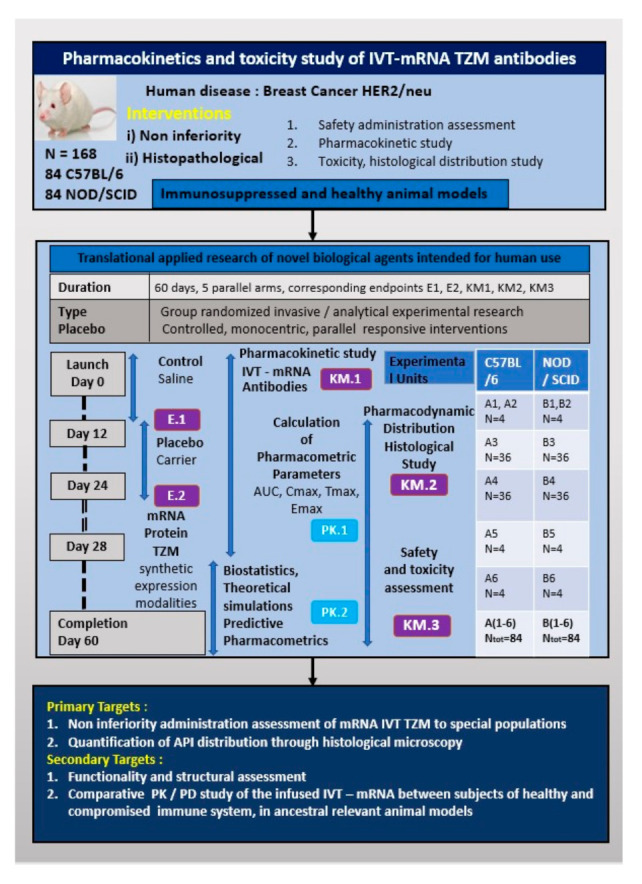
Overview of the pre-clinical trial design points.

The blood samples obtained will be kept in appropriate, sterile tubing and centrifuged in 4 °C for 5 min at 2000× *g*, plasma will be separated and collected and the supernatant will be isolated and documented for each working group and finally stored in deep freezer (−80 °C), conserved for the quantification analysis steps by ELISA and followed by LC-MS validation. The overview of the pre-clinical design is presented by the poster of Figure 2. 

In order to achieve the outmost of the experimental resources and ensure robustness and seamless pre-clinical implementation, reduce future protocol amendments and advance towards the facilitation of the study’s both ethical and scientific considerations appraisal, the SPIRIT statement has been archived to inform all the stages of the trial [41]. In Table S2 of the Supplementary Materials the Schedule of enrolment, allocation, interventions and control assessments, aligned to the SPIRIT guidelines are presented.

### 2.4. Randomization and Blinding 

Each mouse subject will be assigned a randomized block before launching the work packages of the protocol. Given the restricted sample size of our preclinical study, a stratified randomization technique has been extended to include the “Guidelines and Initiatives for Good Research Practice” [42] for all working animal groups, being identified before group assignment. This method implements principles of probability theory [43] in order to assess the likelihood as a source of difference between the delivered outcomes, thus enabling the application of central limit theorem [44] based tests, evaluating the minimum statistically viable animal sample number, utilizing the developmental algorithm found at (https://www.randomizer.org/, accessed on 8 February 2021). Following the proposed arrangement scheme the sample characteristics are maintained homogenous and consistently representative of the typical population. Moreover, the sample may be drawn by any experimental group at any given time point, hence all the animal units receive monodose administrations via intravenous route and with equal pre-treatment handling. Each treatment group will be matched with a separate color mark indicated on its tail. The identical simple to discern coding, will be applied for: (a) Vials containing the experimental drug; (b) controls and placebo; and (c) syringes used to infuse the tested substances.

Following the iterated discrimination and in an effort to achieve satisfaction of the relevant GCP compliant directives, data integrity will be monitored in real time within the active clinical period. Due to the fact that the protocol will be executed stepwise and that the subjects will, at all times, be handled by a limited member team of experienced scientists, a partial blinding during the experimental treatment will suffice and will be followed by a full blinding only during the phases of data collection and analysis [42]. This decision is justified by the fact that the under-investigation drug suspensions are of similar physicochemical feature and appearance, identical viscosity, thus, constituting visual recognition an impossible task.

In relation to such infusions, researchers have questioned the robustness of masking of content ingredients as blinding method, hence, visual inspection is acknowledged as a safety precaution standard, and thus, advocates that the acceptability of this technique should be limited. Therefore, in certain cases similar to our protocol, the deployment of unblinded or partially unblinded handling using color coding, though not perfect, does not lead necessarily to unblinded conditions. Indeed the limiting of the personnel access to that of analyzing treatment results only, offers counter measures balancing against inappropriate unblinding [45].

### 2.5. Statistical Methods and Preliminary Feasibility Report

To assess the minimum sample of murine models required to produce meaningful statistical results, a non-inferiority t-test for two independent means power analysis was conducted by the software nQuery Cork, Ireland, covering the pilar pharmacokinetic experimental groups: (a) The A3 and B3 groups; and (b) A4 and B4 groups, respectively. We have predefined the delta Δ_o_ margin of noninferiority hypothesis limit difference to be 20 ug/mL plasma concentration in order to define the therapeutic interchangeability [46]. The results showed that for sample size in both groups being three, a one-sided two group 0.05 significance level *t*-test has 80.49% power of rejecting the null hypothesis that the standard and test are not non-inferior (the difference in means being 20 or farther from zero in the same direction), in favor of the alternative hypothesis that the means of the two groups are non-inferior. This assumes that the expected difference in means is 0.001 and the common standard deviation is 8.

**Figure 3:**
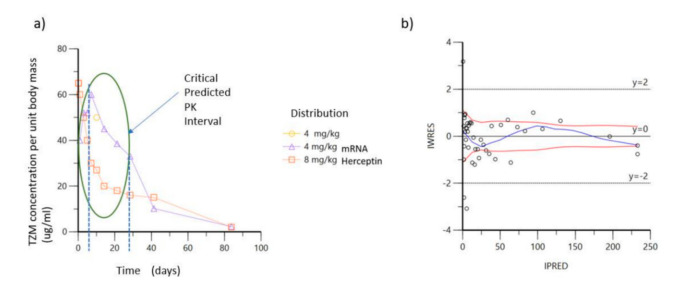
Concentration curves of TZM over time after intra venous infusion: (**a**) Predicted critical time window of interest based on projections of bibliographic data; (**b**) IWRES versus IPRED for predicted data of mAb infusion as full-length protein.

Moreover, based on the existing experimental data and results published by relevant mAb clinical trials in human and animal subjects [47], we attempted to chart a prediction of the expected PK profile of antibody expression, post the intravenous infusion of lipid nanoparticles containing TZM encoded by the mRNA constructs, regarding the A5, B5 and A6, B6 groups. The scenario advocates the restricted measurement of therapeutic antibody concentration for the experimental unit, allowing for almost two-fold reduction of the animal subjects’ number and is presented by Figure 3a.

Elaborating on the former statement, by performing the above preliminary analysis, we were able to restrict the observation of our experimental work plan within the time frame of 60 days and further define the window of dense sampling to focus the chronic period between days 0 and 28, as indicated by the ellipse of Figure 3a. The individual weighted residuals IWRES plotted against individual predicted values (IPRED, predicted concentrations) for the antibody pharmacokinetic profile is demonstrated by Figure 3b, showing that the observed against fitted values fall close to the line of unity, thus, demonstrating the feasibility of our approach.

Due to the modular nature of our design we hereby are able to introduce a parallel, custom statistical observation method presented by Table 2, whereby, subjects are initially taxonomized for each animal type and are stratified according to the weight-based dosing regimen administration for each time interval, delivering a response contour space suitable for scanning the desired levels of TZM systemic bioavailability. 

**Figure 4:**
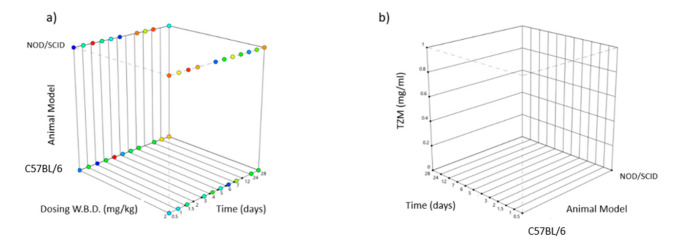
Box diagram showing the experimental design [48] for various factors [49]: (**a**) *y*-axis type of animal model categorical NOD/SCID/J (top) and C57BL/6/J (below), *x*-axis W.B.D. dosing in mg/kg, *z*-axis time in days, (**b**) *y*-axis TZM blood concentration in mg/mL, *x*-axis time in days, *z*-axis type of animal model C57BL/6/J (left) and NOD/SCID/J (right).

The above design is implemented to aid the comprehensiveness of the statistical output translation, hence multiple interventions are studied simultaneously decreasing the participant subject’s number. This design is schematically represented by Figure 4, showing the dual factorial levels being employed for animal model type, namely C57BL/6/J and NOD/SCID/J, binary levels for W.B.D. dosing namely 2 mg/kg and 8 mg/kg and 12 levels for time checkpoints (0.5, 1.0, 1.5, 2, 3, 4, 5, 6, 7, 12, 24, 28) days and with the final response being appointed to the TZM accumulation in blood post-infusion measured in concentration units of mg/mL.

### 2.6. Preparation of Drug API Lead mRNA and Vehicle Formulation 

The lead of mRNA constructs have been hierarchically designed by rational bioinformatic abstraction methods as autonomous functional genetic circuits, i.e., masking physical details [50]. The codon sequences were aligned to the PDB humanized TZM antibody and the BLOSUM62 algorithm was used to define the percentage of differentiation [51]. Compared to the wild type of TZM several technical mutations have been appointed: (a) G (guanine)/C (cytosine) enrichment of the coding sequence (CDS) locus capable of producing stable rich A (adenine)/U (uracil) transcripts based on codon degeneration [52], (b) codon-recognized tRNAs insertions [53], (c) replacement of AURES of 3′ UTR sequences recognized by Toll like receptors and nucleases [54], (d) post-transcriptional capping anti-reverse analog (ARCA), modified nucleotides (5-Methylcytidine-5′-Triphosphate and Pseudouridine-5′-Triphosphate) to enhance the stability and reduce the immune response of host mammalian cells, (d) 3′-PolyA-tail addition to increase translation efficiency of transfected cells by the recruitment of poly(A) binding proteins, conferring also stability by inhibiting exonuclease activity, (e) Kozak sequence (GCCGCCACCAUGG) binding to ribosome translational enhancer, (f) IRES (Ιnternal Ribosome Site) polycistronic mRNA transcriptomic enhancer, (g) UTR stabilizing sequences 5′ of b globulin, (h) secretory signal. In detail, we performed the proof of concept protocol, utilizing T7 RNA polymerase to evaluate the in vitro transcription and potency of mRNA expression of TZM for the transfection of mammalian cell cultures. The latter was used to transcribe the designed linearized DNA template into mRNA, (HiScribe T7 ARCA mRNA Synthesis Kit, NEB Biolabs). Subsequently, the reaction was incubated at 37 °C for 30 min and treated with DNase to remove excess template DNA [55].

**Figure 5:**
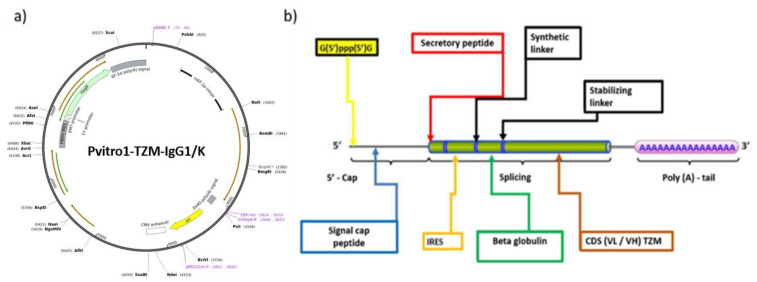
(**a**) Plasmid preparation backbone; (**b**) mRNA optimization chart showing the additions and mutations utilized for our clinical experiment.

The CDS light V_L_ and heavy V_H_ chains were optimized and integrated to the pVITRO1 plasmid backbone (see Figure 5a), based on the latest of lege artis methodology, in an effort to improve stability and translationability of the investigated mRNA modalities. In Figure 5a,b, the plasmid construction and the induced mutations that lead to stability and functionality of our mRNA lead, are revealed. In addition, purification of RNAs from enzymatic synthesis or modification reactions, an essential process prior to mammalian cell transfection, was performed. Expanding on the former statement, upon mRNA synthesis by in vitro transcription, unincorporated nucleotides, short aborted transcripts, small molecule, enzyme reaction and buffer components were removed before formulating the mRNA. For the purification of mRNA, we used the Monarch RNA Cleanup Kit (NEB Biolabs). The Monarch RNA Cleanup Kits provide a fast and simple column-based solution for RNA cleanup and concentration (binding capacities: 10 μg, 50 μg) after in vitro transcription [56]. 

**Figure 6:**
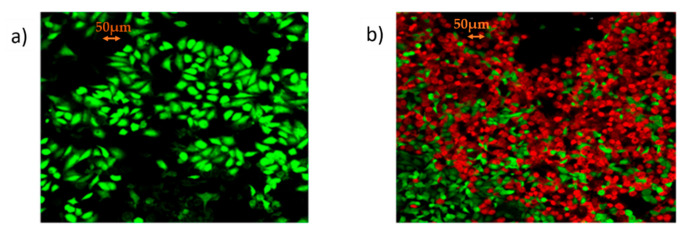
Proof of clinical concept: (**a**) Confocal microcopy microphotograph of MCF7 cells by the live/dead fluorescent staining with Calcein AM/EthD-1; (**b**) confocal microcopy microphotograph by the live/dead fluorescent staining with Calcein AM/EthD-1 showing low levels of cell viability of MCF-7 cells, incubated for 72 h with the supernatant of transfected CHO-K1 with mRNA Trastuzumab (photographs were taken at 10× magnification; scale bars represent 50 μm).

The synthesized mRNA within the lipid carrier (mRNA/jetMESSENGER^®^ ratio 1:2), was eventually used for the transient transfection of Chinese hamster ovary CHO-K1 (Registered Code: CCL-61, Sourced by: ATCC) wild type; American Type Culture Collection, Rockville, MD, USA)) cells while the optimal culture conditions were determined for the biosynthesis of TZM. After 48 h of incubation, transfected CHO-K1 cells were selected out, the cytoplasmic RNA was isolated and assessed by Reverse Transcription-Polymerase Chain Reaction (RT-PCR) analysis, for the steady-state level assessment of TZM RNA transcripts that were encoded by the Heavy and Light Chains, respectively. After 72 h, the culture supernatant of transfected CHO-K1 cells was collected, containing the TZM antibody. MCF7 (Registered Code: HTB-22, Sourced by: ATCCcell line) positive HΕR2+ receptor were incubated with the supernatant of the transfected CHO-K1 colony with DMEM 10% FBS at 37 °C and 5% CO_2_ for 72 h and the evaluation of cell viability was detected by the live/dead fluorescence staining. Cells were finally double stained with Calcein AM and Ethidium Homodimer-EthD1 (live/dead respectively) and were visualized under a confocal microscope (Leica Microsystems, Wetzlar, Germany), as shown by Figure 6a,b.

Conclusively having demonstrated proof of concept, several lipid vehicle nanoparticles (LNPs) shall be prepared towards the pre-clinical trial implementation, containing mixtures of DSPC: Chol: DOTAP: DMGPEG 2000 by individual lipid stocks kept in ethanol. Lipid mixture will be dissolved at 2 mg/mL in ethanol at several quality by design pre-defined molar ratios [57]. Citrate buffer of pH 6 at 100 mM will be exploited to serve for the aqueous dispersion phase. The effect of several coatings (DMG-PEG2000 vs. DSPE-PEG2000), condensing polymeric agents (DDAB, DOTAP or MC3) and structural lipids (HSPC vs. DSPC) will be assessed [58]. For the preparation of the encapsulating mRNA, an aqueous phase of citrate buffer regulated at pH 6, 100 mM and presenting a nitrogen (from the cationic lipid) over phosphate (from the nucleic acid) ratio of 8, shall be prepared [59]. Formulations will be subjected to dialysis for the period of 1 h under magnetic stirring conditions, in 200 mL TRIS buffer maintenance in order to avoid sensitivities against high-salt containing buffers and reduce the solvent residues below the regulatory target of 5000 ppm [59].

### 2.7. Risk Assessment 

**Figure 7:**
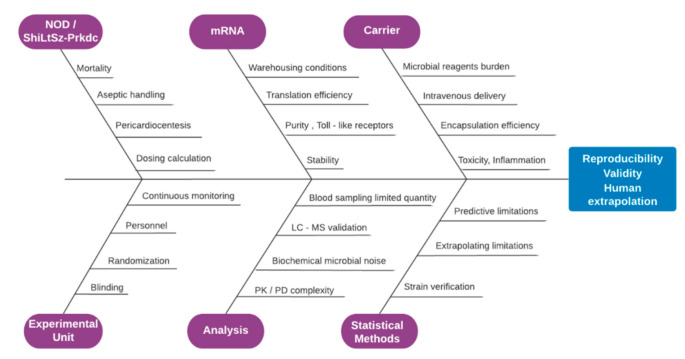
Fish bone diagram of risk assessment demonstrating the factors that critically alternate the validity of the obtained results and effect reproducibility and extrapolation, spanning the sectors of animal type, experimental unit preference, mRNA, analysis methods, carrier and statistical methods employed.

A risk assessment complementary study has been conducted to ensure the credibility of the results by projecting eminent hazards, advice an early action plan and avoid the unethical loss of animals in strict accordance to the ARRIVE guidelines [39], as demonstrated by Figure 7. Combined immune deficiency, spontaneous mutant mice do not acquire functional T-cells and B-cells, while myeloid, antigen-presenting and NK cell functions depend on the selected strain. These homozygotes do not possess Ig- G1, G2a, G2b, G3, A, M, in detectable quantities in their lymph nodes, thymus and splenic follicles, being devoid of any lymphocytic type [30]. Although a limited portion of SCID mice may develop weak immune reactivity spontaneously, leakiness is found in alarming levels in subjects that are housed in facilities that do not support proper SPF conditions. 

Moreover, thymic lymphomas do occur at extremely high frequencies, while the expected life span is limited for these animals to only 8.5 months under pathogen-free conditions [27]. There exist numerous variables that may irrevocably compromise the primary target of our clinical study and constitute the latter a demanding one, namely the: (a) Iterated frail idiosyncrasy of the need based chosen animal model, enhanced by the SPF environment of the clinical setting; (b) intravenous administration in parallel, i.e., simultaneous fashion for all animal groups exposed; (c) medicine development study directives required by the regulatory authorities in compliance to the specific pharmacokinetic rules applied for medicines intended for future use in human; (d) accurate evaluation of the inflammation profile caused by the mRNA or the carrier or their combination after infusion to the blood stream by biochemical characterization; (e) culture-mediated production and special storage conditions for the mRNA modalities when mixed with the carrier; (f) demand that results obtained must for all participating animal groups be reproducible and extrapolatable for human special populations.

Therefore, in order to avoid unacceptable quality failures and mitigate the above-mentioned risks, a cautionary plan regarding the appropriateness of the experimental drug and vehicle formulation, has been apriori integrated to our design. Under this lens, all necessary trial materials in contact with the murine models will be handled aseptically and all relevant substance preparations will be conducted strictly in laminar flow conditions conforming to GMP regulation, taking advantage of the inhouse GLP accredited facility located in the same complex with the clinical setting [60]. All starting material of the carrier and mRNA shall meet the requirements of either pharmaceutical quality grade or at least suitable for veterinary use approaching the analogous biological and chemical purity specification limits posed by the European Pharmacopoeia ruling [61]. A qualified person appointed by the study sponsor and preferably specialized in GMP manufacturing will audit and register the data twice in separate locations and verify that conditions are met before unleashing the clinical batch [62].

### 2.8. Histological Examination and Toxicity Studies

Evaluation of alterations caused by novel drugs to the experimental units of a clinical investigation represents the pilar of any safety assessment before applied to human patients. The latter is based on conventional histopathological tests, representing a significant contribution to novel treatment development for both human and animal diseases [63]. We will evaluate malignant alterations to all organs and their corresponding tissues and investigate the causal relationship that might have been developed due to the novel drug treatment exposure and also assess the relevance that the various adverse effect related findings might have for future patients [64]. For the groups A5, Β5 and A6, Β6 detailed drug biodistribution and toxicity analysis will be conducted followed by the histological examination utilizing fluorescent optical microscopy techniques (DAPI, GFP) exploiting the Bitplane Imaris, ImageJ software to assess signal quantification [65]. For the rest of the groups blood escorting biochemical and hematological tests will be conducted to include the biomarker metrics [66] that are revealed in detail by Table 3.

### 2.9. Data Collection Analysis and Pharmacokinetic Simulations

Compared to small organic APIs, mAbs are characterized by limited transcapillary and tissue permeability, enhanced hepatic metabolism and renal filtration in combination with Brambell receptors salvage, peripheral clearance and nonlinear mass transport caused by receptor binding [67]. The pharmacokinetic study variables and formal data analysis method is summoned by Table 4.

**Figure 8:**
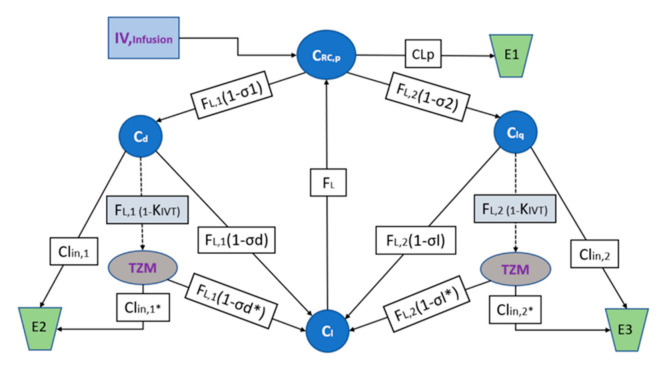
Model structure of the PBPK TZM mRNA IVT for the plasma baseline concentrations. $\mathrm{Whereas}\;C_{\mathrm{RC},P}$ is the mAb mRNA TZM concentration found in plasma volume $V_{P}$, $C_{d}$ and${\;C}_{\mathrm{lq}\;}$are the mAb concentrations in ISF in the tissues dense and leaky of volumes $V_{d}$ and $V_{\mathrm{lq}}$ and of continuous, and fenestrated capillaries, respectively, $V_{l}$ is the lymph volume and assumed almost equal to blood volume. The $F_{L\;}$ is the total lymph flow which is considered equal to the sum of $F_{L,1\;}$and $F_{L,2\;}$ being the lymph flux for $V_{d}$ and $V_{\mathrm{lq}}$ respectively. The $\sigma_{d}$ and $\sigma_{l}$ and the $\sigma_{d*}$ and $\sigma_{l*}$ being the coefficients of vascular reflection $V_{d}$ and $V_{\mathrm{lq}}$ for the mRNA formulation and the correspondent TZM IgG, respectively. The ${\mathrm{CL}}_{P},{\;\mathrm{CL}}_{\mathrm{in},1}$, ${\mathrm{CL}}_{\mathrm{in},1*}$ and ${\mathrm{CL}}_{\mathrm{in},2}$, ${\mathrm{CL}}_{\mathrm{in},2*}\;$are the clearances for the plasma and ISF dense, leaky tissues for the mRNA formulation and the correspondent TZM IgG, respectively. Simulations of the predicted active tissue concentrations will be compared to the experimental measurements in order to evaluate the model performance. The areas under the concentration versus time curves (AUC0-t) will be calculated using the NCA bundle of Phoenix 8.1 (2021, Pharsight Corporation, Mountain View, CA) and predictions will be extrapolated using the same software for the relevant human populations.

Both mAb diffusion through the lipid bilayers and distribution in the tissue interstitial space depend on convection through the pores of the endothelial vasculature and transcytosis. While, endosomal escape plays a critical role by ensuring the ribosomic translation event and the consequent transport to the interstitial space, thus, ameliorating the lysosome degradation processing. Modeling predictions will concern the evaluation of extravascular distribution, i.e., the transcapillary escape rate (TER) and protein localization [68]. A third-generation system-average physiologically based pharmacokinetic (PBPK) model has been considered for the relevant tissue types taking into account the mAb expression prodrug particularities. Moreover, the recent use of high-resolution biosensor technology, combined with advanced numeric tools, has revealed that all interactions, such as those based on monoclonal antibodies are more heterogenously than what has been previously understood. These effects are in fact embedded in the apparent PKPD parameters presented by Table 4 [69]. The model interrogates the translation, convective transport uptake of IgG, endocytosis and organ distribution into three main compartments, i.e., vascular, endosomal, interstitial [70], and is demonstrated by the scheme of the Figure 8.

Targeted-mediated drug disposition (TMDD) and Toll like receptor inactivation will be integrated to the PBPK model coefficients in an effort to estimate the effects of receptor binding in the plasma and the interstitial space and the parameters describing the behavior of endogenous IgG will be experimentally fixed. The model will offer predictions for the mAb concentration against time for the investigated compartment types leaky and tight, and their clearance mechanisms assuming one-pore formalism, convection being the dominant distribution pathway and ISF as the main extravascular distribution space [68]. The algebraic model structure is principally described according to the relevant methods for Pharmacokinetic and Pharmacodynamic Data Analysis as described by J. Gabrielsson and D. Weiner [71] and is schematically appointed by the diagram of Figure 8.

### 2.10. Statistical Analysis

Upon study completion, collection and curation of data, statistical analysis will be implemented using SPSS (IBM Deutschland GmbH, Ehningen, Germany) and Prism GraphPad Software (San Diego, CA, USA).The results shall be considered as significant for *p* values < 0.05 and for the baseline and all efficacy and safety variables the following steps will be undertaken: (a) Individual concentration values will be documented and graphically presented for each experimental unit of each group; (b) descriptive statistics shall be tabulated for each experimental group per time intervals while the mean and standard error will be calculated and plotted; (c) for the discrete values a table of frequencies will be presented over time scale for each group; and (d) comparisons between the intervention treatments and placebos will utilize Student’s *t*-tests, Wilcoxontest ranks for non-parametric data while correlation interactions will be analyzed by Pearson’s coefficient calculation, and Spearman’s test, respectively. A combination of multivariate analysis will be adapted whether appropriate and a mixed effect models shall be considered.

## 3. Discussion

Implementing a multi arm, cohort randomized, premier, double blind placebo-controlled trial design should generate credible data to investigate the safety and non-inferiority of mRNA-encoded TZM antibodies, compared to the marketed substance, in order to aid the development of a first line medication for immunodeficient patients suffering from HER2 -neu breast and gastric cancer. If clinical endpoints are successfully reached and deliver significantly credible statistical results, our experimental efforts would establish the potential to investigate an alternative therapy for special populations, thus, covering an unmet need. This study will utilize a double and partially blinded, placebo-controlled protocol following a parallel design nomenclature. Considering that the designed features encumber the highest of quality preclinical standards, the challenges against the bibliographically known or expected biasing effects have been addressed. Animal participation and sacrifice has been minimized by the utilization of the latest fourth industrial age toolbox, borrowing traits and methods from current relevant human trial design paradigms. The block randomization, the initiation of placebo control, the two-level blinding features are evidently expected to ameliorate both the regression to the mean variance and the anticipated observer artifacts frequently occurring in mice studies [72], especially found when exploring precision medicine intended for future human use.

Such design significantly increases the representativeness and accuracy of results on the application of IVT-mRNA as alternative or first line therapy instead of monoclonal antibodies. Regression to the mean is critical for clinical investigations when variables range to extreme propensities, and when the selected population is either not uniform and/or the induced disease models are applied in uni-linear fashion [73]. In this preclinical study, we leverage on the recruitment of an established, reliable strain of a verified animal model that is borne with the artificial preferred phenotype and housed in clean, continuously monitored conditions [30]. The former in combination with the aseptic starting material and drug preparation, good clinical practice appropriate warehousing and handling advocate the opportunity to produce credible, repeatable results for any chromic interval, randomly chosen for validation [36].

The placebo effect has been established as of outmost importance element in human trial design but in pre-clinical practice it has been honored with limited application so far, especially when the immunology field and particularly for the involvement of mice models [74]. For the proposed implementation partum pendo, each experimental unit serves as if being the same organism, receiving treatment or control and being biochemically and physiologically tested at several points in time. In other words, this pre-clinical study presents an unusual “between patient”, “non-crossover” protocol of antibody testing, according to which distinct arms of treatment are administrated, such as the first group receives treatment arm A as pro drug and the alternate group receives treatment arm B drug as comparator [75]. Our two-parallel intervention treatments are composed of completely different formulations, doses, API chemistries and mechanism of action of the same principal drug substance. The advantage of this parallel design is the combined safety and non-inferiority assessment of the experimental drug as the critical endpoint of evaluation. 

Furthermore, we incorporated the available internal validity criteria preached by the “Guidelines and Initiatives for Good Research Practice” [36], addressing the issues of randomization blinding. Both randomization and blinding interact with additional confounding variables in order to achieve reduction of bias risk [72]. Under this lens and taking into account our sample size we employed a tailored pseudo-randomization protocol based on a simple algorithm that can be applied repeatably by the personnel, thus ensuring that: (a) Experimental unit assignment is equal for each of the subjects; and (b) each assignment to any of the groups cannot affect the inclusion of any other subject to the same group. The subjects were allocated to either treatment or placebo conditions, maintaining the randomization state throughout the study conduct to avoid primary and secondary outcome performance bias.

Risk assessment analysis was applied, in order to decide the fit-for-purpose blinding type of for the specific study conditions, according to the ICH harmonization Principles of “Evidence-Based Medicine to Optimize Research Practices” ruling [76]. Blinding was justified as tailored by the needs of the specific experiment and balanced against the existing standard operating procedures and the potential risks. For our clinical trial during the experimental treatment and the collection of biological samples, discrete color coding allows the efficient recording, so as full blinding will be implemented only when deemed necessary, i.e., during the analysis and interpretation steps [45]. Reliable results will be received following this pathway and avoiding the imminent complexity drawbacks of over blinding measures whereas our personnel will be required to handle vials that are beforehand presented as identical container units to any observer. Our non-inferiority trial incorporates a placebo and comparator, thus, we are pursuing multiple goals. We seek to establish superiority to placebo, validate the experimental design and at the same time evaluate the similarity of safety and efficacy to the comparator. Therefore, this trial cannot be classified as a typical equivalence venture hence it is not mission-conservative. 

Unchartered complexities and design flaws or conduct misfits will certainly bias the results. Consequently, special care has been given to acknowledging, registering and where possible minimizing the associated risks and related violations of inclusion criteria, withdrawals, non-compliance, missing data, protocol and subsequent analysis deviations. The above are linked to several limitations of our study that may reflect negatively on the proper translation and extrapolation of data, such as the intention to treat analysis (ITTA) may be challenging or impossible to perform [77]. Given the experimental unit is assumed as the singular investigated organism, where randomization is not successfully implemented, false AUC values may be incorporated to the statistical analysis describing an artifact PK, thus, leading to misfit of the extrapolation strategy. Moreover, the need for aseptic starting material and aseptic handling during the preparation and administration stage respectively, increase the trial implementation difficulty level, approaching in certain aspects those of human experimentation.

## 4. Conclusions

We performed non-inferiority pharmacokinetic studies to evaluate the safety and the curve of dose response exposure, prior to a detailed pharmacodynamics and efficacy assessment for IVT encoded TZM mAbs. Plasma and tissues will be collected and examined biochemically and histopathologically over the selected time milestones, in order to interpret the AUC results obtained. Further theoretical assessment of raw data will be approximated by PBPK modelling in an effort to explain and extrapolate the results for human special populations in need. To our knowledge, this is the first clinical trial designed to provide in-depth, the comparative pharmacokinetic and pharmacodynamic impact of mRNA-encoded monoclonal antibody vaccinations on immunodeficient organisms and meaningful data regarding safety and efficacy will be obtained. 

Taking into account that the mRNA lipoparticle-based vaccinations against SARS-CoV-2 have achieved 95% efficacy [78], and that a Phase 1 tolerability study has been recently completed in healthy human adults, determining the dosing regime and pharmacodynamics of mRNA antibody expression against Chikungunya virus (6/2021, https://clinicaltrials.Gov/ct2/show/NCT03829384?term=mRNA-1944&rank=1,accessed on 2 August 2021), more adaptive pre-clinical to bedside trial designs towards the development of novel immune RNA medicines are urgently needed. Moreover, after the recent FDA emergency authorization, for clearing the use of monoclonal antibody administration also to immunosuppressed COVID-19 diagnosed patients presenting mild-to-moderate virus burden [79], the potential of the said therapies is upgraded. The development of additional monoclonal antibody modalities to meet the iterated demand is intrinsically linked to novel, reliable, pre-clinical development strategies, effectively implemented in the limited time-given work frame. Our study protocol contributes to this purpose by offering an elaborate method statement, one to guide the pre-clinical assessment of mRNA encoded monoclonal antibodies with special focus on their unmet application to immunosuppressed populations.

## Figures and Tables

**Table 1:** Intervention schedule for each animal type according to dose regime and substance administrated for the time monitoring checkpoints.

Group	Type	Intervention	Route	Mice (*n*)	W.B.D. Regime (mg/Kg)	Dose (μL)	Monitoring Intervals (Days)
A1	C57BL/6/J	Saline Solution	Iv	2	-	100	12
B1	NOD/SCID/J	Saline Solution	Iv	2	-	100	12
A2	C57BL/6/J	Placebo	Iv	2	-	100	12, 24
B2	NOD/SCID/J	Placebo	Iv	2	-	100	12, 24
A3	C57BL/6/J	Trastuzumab (mRNA + Carrier)	Iv	36	2	40	0.5, 1.0, 1.5, 2, 3, 4, 5, 6, 7, 12, 24, 28
B3	NOD/SCID/J	Trastuzumab (mRNA + Carrier)	Iv	36	2	40	0.5, 1.0, 1.5, 2, 3, 4, 5, 6, 7, 12, 24, 28
A4	C57BL/6/J	Trastuzumab (Protein)	Iv	36	8	160	0.5, 1.0, 1.5, 2, 3, 4, 5, 6, 7, 12, 24, 28
Β4	NOD/SCID/J	Trastuzumab (Protein)	Iv	36	8	160	0.5, 1.0, 1.5, 2, 3, 4, 5, 6, 7, 12, 24, 28
A5	C57BL/6/J	mRNA GFP	Iv	4	2	40	6, 18, 24, 28
B5	NOD/SCID/J	mRNA GFP	Iv	4	2	40	6, 18, 24, 28
A6	C57BL/6/J	GFP (Protein)	Iv	4	8	160	6, 18, 24, 28
B6	NOD/SCID/J	GFP (Protein)	Iv	4	8	160	6, 18, 24, 28

**Table 2:** Stratification of the animal model and dosing levels W.B.D. over time to search the design space for the optimum bioavailability.

Factor	Name	Units	Type	Minimum	Maximum		
A	Animal Model		Categoric	C57BL/6/J	NOD/SCID/J	Levels:	2
B	Dosing W.B.D.	mg/Kg	Categoric	2	8	Levels:	2
C	Time	days	Categoric	0.5	28	Levels:	12

**Table 3:** Toxicity and inflammation effects of interventions to be measured by the various parameters of serum.

**Effects of Interventions to be Measured on the Various Parameters of Serum**							
Leukocytes	BA	EO	MO	LY	NE	WBC	
Erythrocytes	RDW	MCHC	MCH	MCV	HCT	Hb	RBC
Thrombocytes	MPV	PLT					
Miscellaneous	Anion Gap	Phosphorus	Sodium	Potassium	Bicarbonate	Calcium	BUN
	Total Bilirubin	Globulin	Cholesterol	Alk Phos	AST	GGT	ALT
	CK Bilirubin Unconjugated	BUN/Creatinine	Chloride	Bilirubin Conjugated	Creatinine	Glucose	CK
	Total Protein	ALB/GLOB	Albumin		NA/K		

**Table 4:** Study parameters and relevant data will be collected and monitored by the investigator team assessing the PK/PD effects. The following table provides an overview of the planned PK variables and their corresponding calculation methods.

Measuring Points	E.1, KM.1	E.2, KM.1	PK.1, KM.2	PK.2, KM.2, KM.3
Study Day	D 0–12	D 24	D 28	D 60
Study variable	Variable type		Variable Description	Unit
	**Outc.**	**Covar.**		
**Demographics**				
Body weight		X	Subjects mass	gr
Age		X	Age classification	months
Temperature		X	Body temperature measurements	°C
**Dose (D)**			Amount of drug to be administered	mg/kg
AUC	X		Total area under the plasma drug concentration	(μg × h/mL)
AUMC	X		Total area under the first moment curve	(μg × h^2^/mL)
**Drug conc.**				
A, B	X		Coefficients of biexponential equation	-
Cp_(0)_	X		Initial drug concentration in plasma	mL/h
Cp_(12 h)_	X		Plasma drug concentration at 12 h	mL/h
Cp_(last)_	X		Last measured plasma drug concentration	mL/h
C_p,ss_	X		Plasma drug concentration at steady-state	mL/h
C_p,ss(max)_	X		Maximum desirable plasma drug concentration	mL/h
C_p,ass(min)_	X		Minimum effective plasma drug concentration	mL/h
C_p(avg)_	X		Average plasma concentration	mL/h
C_max_	X		Peak concentration of drug in blood plasma	mL/h
C_min_	X		Minimum concentration of drug in blood plasma	mL/h
**Time notations**				
t_1/2_	X		Half-life	h
t_1/2(a)_	X		Absorption half-life	h
t_1/2(d)_	X		Apparent half-life	h
MRT	X		Mean residence time	h
MAT	X		Mean absorption time	h
t_max_	X		Time at which peak plasma concentration occurs	h
**Rate constants**				
a, b	X		Exponents of biexponential equation	-
k_12_,k_21_	X		First-order rate constants	-
k_el_	X		First-order rate constant for elimination	-
k_a_	X		Apparent first-order absorption rate constant	-
k_d_	X		Apparent first-order disposition	--
**Volume terms**				
V_d_	X		Apparent volume of distribution based on AUC	L/kg
V_d(ss)_	X		Apparent volume of distribution at steady-state	L/kg
V_c_	X		Apparent volume of pharmacokinetic model	L/kg
**Clearance**				
Cl_B_	X		Body (systemic) clearance	mL /h kg
C_lR_	X		Renal clearance	mL /h kg
C_lH_	X		Hepatic clearance	mL /h kg
Q_organ_	X		Clearance expressed on unit body weight basis	mL /h kg

## Data Availability

Not applicable.

## Supplementary Materials

The following are available online at https://www.mdpi.com/article/10.3390/vaccines9080890/s1, Table S1: SPIRIT checklist.
